# Combined hemophagocytic syndrome and thrombotic microangiopathy due to mixed infection with influenza virus and pneumococcal pneumonia

**DOI:** 10.1002/ccr3.1842

**Published:** 2018-11-22

**Authors:** Takashi Ishiguro, Ayako Kojima, Taisuke Shimizu, Norikatsu Mita, Seiichiro Kuroiwa, Noboru Takayanagi

**Affiliations:** ^1^ Department of Respiratory Medicine Saitama Cardiovascular and Respiratory Center Saitama Japan; ^2^ Department of Nephrology Saitama Cardiovascular and Respiratory Center Saitama Japan; ^3^ Department of Anesthesiology Saitama Cardiovascular and Respiratory Center Saitama Japan; ^4^ Department of Clinical Engineering Saitama Cardiovascular and Respiratory Center Saitama Japan

**Keywords:** atypical hemolytic‐uremic syndrome, hemophagocytic syndrome, hyperinflammatory lymphohistiocytosis, influenza, *Streptococcus pneumoniae*, thrombotic microangiopathy

## Abstract

Development of hemophagocytic syndrome and thrombotic microangiopathy due to community‐acquired pneumonia is rare, but delayed management of these complications can lead to a poor prognosis. Infection by both *Streptococcus pneumoniae* and influenza virus can cause these complications; thus, physicians should pay attention to them when treating influenza‐associated pneumococcal pneumonia.

## 1 INTRODUCTION

Both *Streptococcus pneumoniae* and influenza virus are important pathogens of community‐acquired pneumonia that frequently combine to cause mixed infection. Recently, we experienced a patient who developed hyperinflammatory lymphohistiocytosis (HLH)/hemophagocytic syndrome (HPS) and thrombotic microangiopathy (TMA) induced by community‐acquired pneumonia due to mixed infection with *S pneumoniae* and influenza virus. To our knowledge, only 3 cases^1^, ^2^, ^3^ of combined HLH/HPS and TMA have been reported, and there is only a single published report of TMA induced by pneumonia due to mixed infection with *S pneumoniae* and influenza virus.^4^

## 2 CASE REPORT

A 65‐year‐old man developed dyspnea on effort and general fatigue in March 2018, followed 3 days later by a fever of 38ºC and appetite loss. He had not complained of cough, sputum, or myalgia. Beginning 4 days after the initial symptoms, the patient found it difficult to walk, and he was transferred to our hospital.

The patient had smoked 10 cigarettes per day from age 18 to 63 years and was diagnosed as having the chronic obstructive pulmonary disease. He also had a history of resection of lung cancer 2 years before presenting to our hospital. He drinks one glass of beer per day and has never been exposed to dust nor vaccinated for influenza or *S pneumoniae* infections.

**Figure 1:**
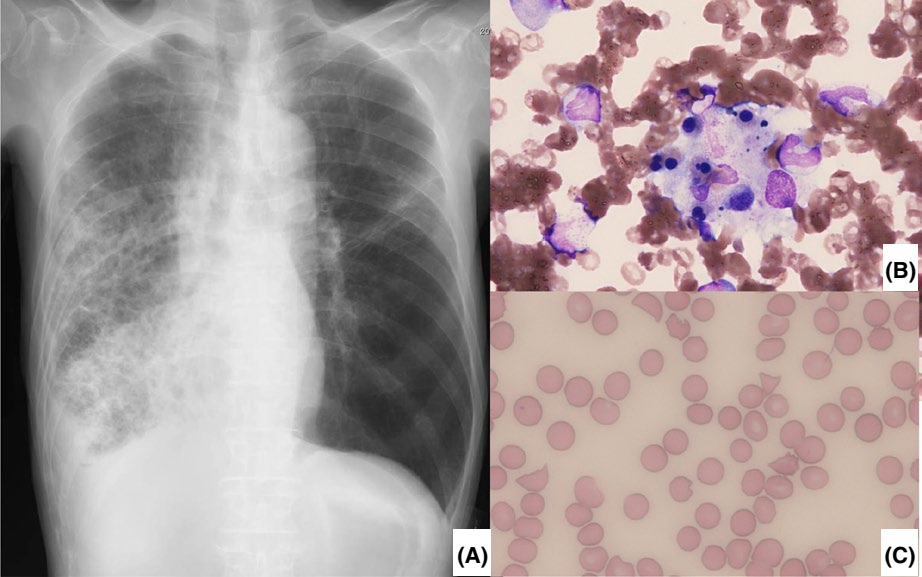
(A) Chest X‐ray on admission showed consolidation in the right lung field. (B) Photograph of bone marrow revealed marked hemophagocytosis. (C) Peripheral blood smear in which fragmented red blood cells were found

On admission, his Glasgow Coma Scale score was E3V4M4 and his vital signs were body temperature 35.1ºC, heart rate 137 bpm, blood pressure 70/30 mmHg, respiratory rate 34/min, and SpO_2_ 78% (under O_2_ inhalation at 10 L/min). Auscultation did not reveal any murmurs, but the air sounds in his right lung fields were attenuated. Blood gas analysis under O_2_ inhalation at 10 L/min showed a pH of 7.36, the partial pressure of arterial oxygen of 69.3 Torr, partial pressure of arterial carbon dioxide of 32.8 Torr, bicarbonate of 18.2 mmol/L, base excess of –6.3 mmol/L, and lactate of 5.75 mmol/L. Peripheral blood tests showed a white blood cell count of 1200/mm^3^ (neutrophils 86.1%, lymphocytes 10.4%, eosinophils 0%, basophils 0.9%, monocytes 2.6%), hemoglobin of 11.8 g/dL, and platelets of 12.8 × 10^4^/mm^3^. Serum biochemistry and serology tests were as follows: aspartate aminotransferase 55 IU/L, alanine aminotransferase 19 IU/L, lactate dehydrogenase 213 IU/L, total protein 6.3 g/dL, albumin 2.5 g/dL, total bilirubin 4.0 mg/dL, blood urea nitrogen 27 mg/dL, creatinine 1.11 mg/dL, sodium 137 mmol/L, potassium 3.8 mmol/L, chloride 102 mmol/L, C‐reactive protein 29.7 mg/dL, procalcitonin 45.91 ng/mL, β‐d‐glucan <11 pg/mL, and soluble interleukin‐2 receptor 2820 U/mL. Rapid urinary antigen test for *S pneumoniae* was positive, but rapid influenza diagnostic test, urinary antigen test for *Legionella* sp, and *Mycoplasma* antigen test using nasopharyngeal swabs were all negative. Sputum and blood culture yielded *S pneumoniae*. Chest X‐ray showed consolidation in the right lung field (Figure 1A).

Our patient's CURB‐65 score^5^ was 5 and we diagnosed him as having severe community‐acquired pneumonia and started invasive pulmonary ventilation in the ICU. Cefepime, azithromycin, immunoglobulin, and recombinant thrombomodulin were administered. His blood pressure did not improve by fluid replacement rehydration, and noradrenaline and vasopressin were started but failed. Because he was in septic shock, we initiated polymyxin B‐immobilized fiber column hemoperfusion, but he developed anuria. Sputum collected on hospital day (HD) 3 yielded *S pneumoniae,* but other significant pathogens were not isolated, so we changed antibiotics to penicillin G. Platelets were reduced to 6000/mm^3^, and his serum ferritin value was elevated to 10 758 ng/mL. Bone marrow aspiration test showed an image of hemophagocytosis (Figure 1B), and we diagnosed him as having HLH/HPS and started corticosteroid therapy. On HD 6, his blood pressure improved, but his serum bilirubin value had increased by 20.3 mg/dL. Abdominal ultrasound test showed neither an ectatic bile duct nor edematous gallbladder. Anemia progressed to a hemoglobin of 6.7 g/dL, and anuria and hyperbilirubinemia indicated TMA. We checked blood smears and found fragmented red blood cells (RBCs) (Figure 1C). On the basis of a decrease in ADAMTS‐13 (a disintegrin‐like and metalloproteinase with a thrombospondin type 1 motif, member 13) activity to 0.488 (normal range: 0.78‐1.57) and serum haptoglobin of <10 mg/dL, we diagnosed him as having TMA. The patient had not developed diarrhea from admission, and plasma exchange was then started. The number of fragmented RBCs (per 1000 RBCs counted) in peripheral blood smears showed zero from HDs 1 and 2, but 30 cells on HD 3, 50 cells on HD 15, 94 cells on HD 17, 42 cells on HD 24, and 24 cells on HD 29. Urine output improved to 400 mL/day on HD 29, and serum bilirubin value recovered to 0.9 mg/dL on HD 39.

The patient had developed ventilator‐associated pneumonia on HD 9, and we switched antibiotics from penicillin G to cefepime. His respiratory and circulatory conditions improved, and he was extubated on HD 24. However, the patient underwent a tracheotomy the next day because he could not fully expectorate sputum. From HD 29, the patient suffered from pneumonia due to *Pseudomonas aeruginosa*. We administered antibiotics according to antibiotic sensitivity testing, but antibiotic‐resistant *P aeruginosa* was repeatedly isolated, and the patient ultimately died from hospital‐acquired pneumonia on HD 43. Paired sera on admission and serum obtained on HD 7 showed a significant increase in antibody titers of influenza B virus (from 10 to 40 titers), and we ultimately diagnosed the patient as having mixed viral and bacterial pneumonia due to influenza virus and *S pneumoniae*.^6^

## 3 DISCUSSION

HLH/HPS is a reactive disorder of the mononuclear phagocytic system characterized by inappropriate histiocytic proliferation and hemophagocytosis in peripheral blood and bone marrow. HLH/HPS is classified into primary (congenital) and secondary types. Causes of secondary HLH/HPS include viral infections such as herpes viruses, adenovirus, and influenza, parainfluenza, measles, and rubella viruses, but microorganisms other than viruses can also cause HLH/HPS.^7^ Rapid influenza diagnostic test results in our patient were negative, but both urinary antigen test and culture were positive for *S pneumoniae*. Paired sera showed a significant increase in antibody titers for influenza B virus, and thus, we could not clarify which pathogen was the cause of HLH/HPS.

We started corticosteroid therapy for HLH/HPS and although blood cell counts increased, anemia and thrombocytopenia redeveloped. We suspected TMA due to thrombocytopenia, anemia, acute kidney injury (anuria), and hyperbilirubinemia, and additional tests led to the diagnosis of TMA. As an entity, TMA includes, among others, microangiopathic hemolytic anemia and thrombocytopenia with hyaline thrombi mainly comprised of platelet aggregates in the microcirculation, and various degrees of end‐organ failure. Thanks to a better understanding of the etiology and pathophysiology of TMA, we now realize that conditions such as hemolytic‐uremic syndrome and thrombotic thrombocytopenic purpura are also involved.

*Escherichia coli* bowel infection is a major cause of pediatric TMA. Although we did not investigate Shiga toxin, our patient did not develop abdominal symptoms including diarrhea.^8^ However, both *S pneumoniae* and influenza virus are known causes of TMA. Neuraminidase, which *S pneumoniae* produces, acts on the glycoprotein expressed on the surface of RBCs, platelets, and glomerular endothelial cells, and exposes a Thomsen‐Friedenreich antigen (T‐antigen). Plasma includes anti‐T IgM antibodies, which react with T‐antigen to cause aggregation, hemolysis, and endothelial cell damage that result in TMA.^9^ However, anti‐T IgM antibodies are cold antibodies, and a previous report questioned the effect of these antibodies in the human body.^10^ TMA develops 3 to 13 days after invasive pneumococcal infection in 0.4% to 0.6% of cases. Plasma transfusion should be avoided when treating TMA because it includes anti‐T IgM antibodies.

Influenza virus is also a known cause of TMA. Most of the reported patients with influenza who developed TMA suffered from influenza A, with only 3 cases of TMA due to influenza B reported.^11^, ^12^, ^13^ Although mechanisms by which influenza virus induces TMA are unclear, speculation is as follows: (a) neuraminidase is produced by viruses that cause TMA by a mechanism similar to that of *S pneumoniae*, (b) virus antigens expressed on endothelial cells trigger apoptosis, and (c) platelets are activated directly by influenza viruses.^11^, ^14^ Treatment of TMA induced by influenza includes oseltamivir, eculizumab, and plasma exchange.^11^, ^14^ Our patient's rapid influenza diagnostic test on admission was negative, but we based our diagnosis of influenza on the significant increase in antibody titers. We did not administer either neuraminidase inhibitors, because 2 weeks had passed from onset to the diagnosis of influenza, or eculizumab, for fear of increasing our patient's risk of infection. The treatment choice most frequently selected in influenza‐associated TMA is plasma exchange, whose effectiveness has been reported.^14^

As there is only a single previous case report^15^ of TMA due to mixed infection of influenza virus and *S pneumoniae*, the important question was how to treat our patient. T‐antigen on the RBCs of the reported case was positive, and treatment with antibiotics, continuous venous filtration, peritoneal dialysis, and transfusion of washed RBCs and platelets resulted in recovery. Antibiotics against *S pneumoniae* did not improve platelets counts in our patient; rather, his serum bilirubin value increased, and anuria developed late after admission. The platelet count, number of fragmented RBCs, and serum bilirubin value gradually improved by continuous hemodiafiltration and plasma exchange. Although we did not investigate T‐antigen activity on our patient's RBCs, plasma exchange appeared to be effective. The Coombs test is positive in 90% of patients with TMA due to *S pneumoniae* infection,^12^ however, because the test was negative in our patient, we consider influenza rather than *S pneumoniae* infection to be the cause of our patient's TMA.

Our patient developed both HLH/HPS and TMA due to mixed infection by influenza virus and *S pneumoniae*. Mechanisms underlying the development of these two conditions include hypercytokinemia due to HPS, free radicals produced by activated monocytes and macrophages by cytokines. Our patient's elevated serum bilirubin value on admission and fragmented RBCs noted on HD 3 indicated the onset of TMA in the early phase of pneumonia. The number of patients with combined HLH/HPS and TMA appears to be limited. Because each microorganism is known individually to cause these complications, it thus seems natural that these complications would also develop in patients with mixed infection of *S pneumoniae* and influenza virus. It is unclear whether the frequency of these complications is underestimated or if they are truly rare. Nevertheless, because mixed infection with influenza virus and *S pneumoniae* is common,^16^ physicians should pay attention to the development of HLH/HPS and TMA when treating these patients.

## CONFLICT OF INTEREST

The authors have no conflict of interests to declare.

## AUTHOR CONTRIBUTIONS

TI: is the guarantor of the paper, taking responsibility for the integrity of the work as a whole, from inception to published article. AK, TS, NM, SK, and NT: aggregated the data, created the figure, and helped draft the discussion of the manuscript.
